# Clinicoradiological course in coronavirus disease-19 (COVID-19) patients who are asymptomatic at admission

**DOI:** 10.1259/bjro.20200033

**Published:** 2020-07-31

**Authors:** Arshed Hussain Parry, Abdul Haseeb Wani, Mudasira Yaseen, Naveed Nazir Shah, Khurshid Ahmad Dar

**Affiliations:** 1 Department of Radiodiagnosis, Sher-i-Kashmir Institute of Medical Sciences, Srinagar, Jammu & Kashmir, India; 2 Department of Radiodiagnosis, Government Medical College, Srinagar, Jammu & Kashmir, India; 3 Department of Anesthesiology and Critical Care Medicine, Sher-i-Kashmir Institute of Medical Sciences, Srinagar, Jammu & Kashmir, India; 4 Department of Respiratory Medicine, Chest Disease Hospital, Srinagar, Jammu & Kashmir, India

## Abstract

**Objective:**

The study aimed to describe the clinical and imaging course of reverse transcriptase polymerase chain reaction) confirmed coronavirus disease (COVID-19) patients who are asymptomatic at admission.

**Methods:**

This was a retrospective observational study. Severe acute respiratory syndrome coronavisrus-2 (SARS-CoV-2) positive cases that were asymptomatic at admission were retrospectively enrolled. Specific clinical information, laboratory test results, chest CT imaging features and outcome data during hospital stay were collected and analyzed.

**Results:**

137 non-consecutive asymptomatic patients with reverse transcriptase polymerase chain reaction confirmed COVID-19 were enrolled in the present study. On admission, patients had no symptoms but chest CT findings were present in 61/137 (44.5%). Ground glass opacity (48, 78.7%) followed by ground glass opacity with crazy-paving pattern (9, 14.7%) were the commonest type of opacities with posterior, peripheral predominance and lower zone predilection. Among the initial CT positive group of 61 patients, follow-up imaging revealed progression of pulmonary opacities in 13/61 (21.4%), complete resorption in 21/61 (34.4%), partial resolution in 22/61 (36%) and no change in 5/61 (8.2%). The patients in progression group (54 ± 19.7 years) were older and had higher frequency of co-morbidities (46.2%) compared to the other three groups (10.4%). The patients in progression group had a significantly higher C-reactive protein, higher lactate dehydrogenase and lower lymphocyte count than the other groups (all *p*-values < 0.05). The duration of hospital stay was longer in the progression group (27.1 ± 11.4 days) compared to the other three groups (16.12 ± 5.8) (*p* =< 0.05).

**Conclusion:**

Nearly half of the asymptomatic cases with confirmed COVID-19 had abnormal chest CT imaging. Asymptomatic infections can have a variable clinicoradiological course. Clinically, some recover without developing symptoms, some present few mild symptoms whereas some deteriorate. Similarly, imaging follow-up may reveal resolution (partial or complete), progression or no change.

**Advances in knowledge:**

Clinicoradiological course of asymptomatic COVID-19 cases is diverse.

## Introduction

Coronavirus disease 2019 (COVID-19) cases were first reported from Wuhan, Hubei province of China towards the end of 2019 and spread rapidly across the globe with a sustained human-to-human transmission. According to the situation report-136 of World Health Organization (WHO), COVID-19 has rapidly spread across the world, infecting 6.4 million people and causing 382 867 deaths. India has reported 216,919 cases with 6075 deaths as of June 4, 2020.^1^

The causative organism is a novel enveloped single-stranded RNA betacoronavirus known as severe acute respiratory syndrome coronavirus 2 (SARS-CoV-2).^2^ The main symptoms of COVID-19 are fever, cough, fatigue, myalgia, expectoration, shortness of breath and sore throat.^3^ Other less common symptoms attributable to gastrointestinal tract are anorexia, nausea, vomiting, abdominal pain, diarrhea and mesenteric ischemia.^4,5^ Symptoms of neurological dysfunction have also been reported with headache, anosmia, dysguesia, dizziness, altered sensorium and seizures being the commonly reported symptoms.^6^

Many infected patients are asymptomatic. The frequency of asymptomatic infections has been reported in the range of 19–56%.^7,8^ Arons et al reported a high frequency (56%) of asymptomatic infections at the time of diagnosis in their cohort.^9^ These infected asymptomatic patients known as “asymptomatic carriers or covert transmitters,” represent a potential contagious source of SARS-CoV-2, as they unknowingly transmit the infection to others.^10,11^ It is essential to have in-depth knowledge about these asymptomatic or mild symptomatic cases for formulating strategies for epidemiological control of COVID-19. The high infective potential of asymptomatic cases supports the case of wide use of face masks by the general public especially in crowded places to contain the spread of disease. This holds more value in congregate living conditions like old age homes, prisons, orphanages, inpatient hospitalized patients, mental health facilities where many people with fragile immune systems live together.^12,13^

The aim of this endeavor was to study the clinicoradiological course in reverse transcriptase polymerase chain reaction (RT-PCR) positive patients who were asymptomatic at the time of admission in order to understand the clinical course, temporal course of imaging findings and the final outcome.

## Methods and materials

### Cases and study design

This was a retrospective observational study conducted at a designated COVID-19 Care Centre in Kashmir, India. Institutional review board (IRB) approval was obtained. The requirement for patient’s informed consent was waived. RT-PCR confirmed non-consecutive COVID-19 patients who were asymptomatic, diagnosed from March 21 to June 14, 2020 were enrolled. CT was done on the following grounds: (a) previous reports describing positive imaging findings in asymptomatic cases^7^ (b) previous reports describing asymptomatic carrier transmission^9,10^ (c) to understand the behavior of the virus and response of our population in view of divergent courses of disease in different ethnic populations. CT parameters were optimized to minimize radiation exposure to the patients.

Patients who had a positive initial CT at the time of admission and one or more follow-up CT during the hospital stay were included in the final study. The demographics like age, gender, history of exposure/travel, clinical data including symptoms, comorbidities, laboratory results, chest CT findings, clinicoradiological course and outcome data during the hospital stay were collected and analyzed retrospectively.

### CT acquisition protocol and image analysis

CT scans were performed on 16-row multidetector CT scanner (SOMATOM, Emotion; Siemens, Erlangen, Germany). Patients were set-up in a head-first supine position in the CT gantry and scans were obtained in a single breath-hold in a caudocranial direction starting from below the level of inferior end of costophrenic angle up to the thoracic inlet. Scanning parameters used were: slice thickness 1–1.5 mm, tube voltage 100–120 kVp, tube current of 90-130 mAs and a beam pitch of 1.5. The Automatic Exposure Control (AEC) system was used to minimize the radiation exposure to the patients. Images were reconstructed using reconstruction increment of 0.7 mm into a slice thickness of 1 mm. The images were viewed in lung window settings (width of 1200–1600 HU and level of −600 HU) and mediastinal window (width of 400 HU and level of 40 HU).

The CT images were independently assessed by two experienced radiologists who were blinded to the clinical data. Any disagreements between the interpreting radiologists were resolved by discussion and consensus. The following CT imaging characteristics were studied: (a) presence or absence of lung opacities (b) distribution of lung opacities: single lung (left, right lung) or bilateral lungs; (c) location of pulmonary opacities: peripheral, central or both; (d) number of lobes affected; (e) type of the lung opacity: ground glass opacity (GGO), consolidation, crazy-paving pattern, reticulation, halo sign, reverse halo sign, nodules (f) additional signs like air bronchogram sign, bronchial wall thickening, bronchial dilatation, air bubble sign and segmental or subsegmental vascular enlargement; (g) extra pulmonary findings like pleural thickening, pleural effusion, pericardial effusion and mediastinal or hilar lymphadenopathy.

Lung opacities were categorized using Fleischner society glossary of terms for thoracic imaging.^14^ GGO was defined as hazy pulmonary opacity that did not obscure underlying bronchial and vascular structures; consolidation was defined as a pulmonary opacity with non-visualization of bronchial and vascular structures; reticulation was defined as a collection of numerous thin lace-like opacities; halo sign was defined as a ground-glass haze surrounding a nodule or mass; crazy-paving pattern represents thickened interlobular septa and intralobular lines on the background of GGO, resembling pavement stones.

### Statistical analysis

Data were analyzed using the Statistical Package for the Social Sciences (SPSSInc. Chicago, IL, v. 21.0) and Open source epidemiologic statistics for public health (EPI; Dean AG, Sullivan KM, Soe MM, MIT). Mean value and standard deviation was used to express continuous variables whereas counts and percentages were used to express categorical variables. Fisher’s exact test was used to compare the categorical variables and the two sample Student’s *t* test was used for comparison of continuous variables. A *p*-value less than 0.05 were considered statistically significant.

## Results

Of 137 patients enrolled 85 (62%) were males and 52 (38%) were females. All patients had a history of travel to a high risk zone or history of contact with an infected patient. All the patients were asymptomatic at admission. The mean age of patients was 43.1 ± 17.2 years old.

**Figure 1.**
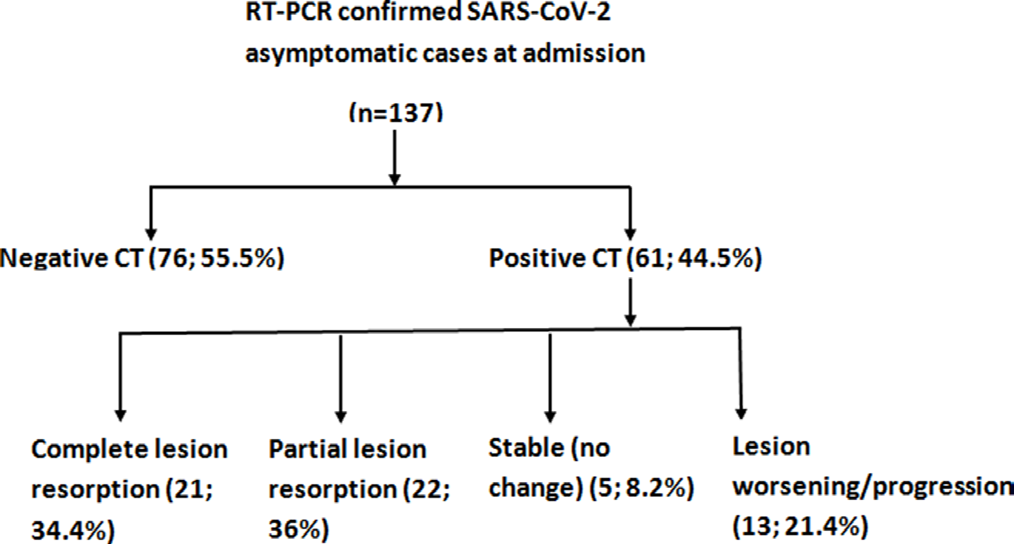
Flowchart depicting the study cohort with their chest CT results.

**Figure 2.**
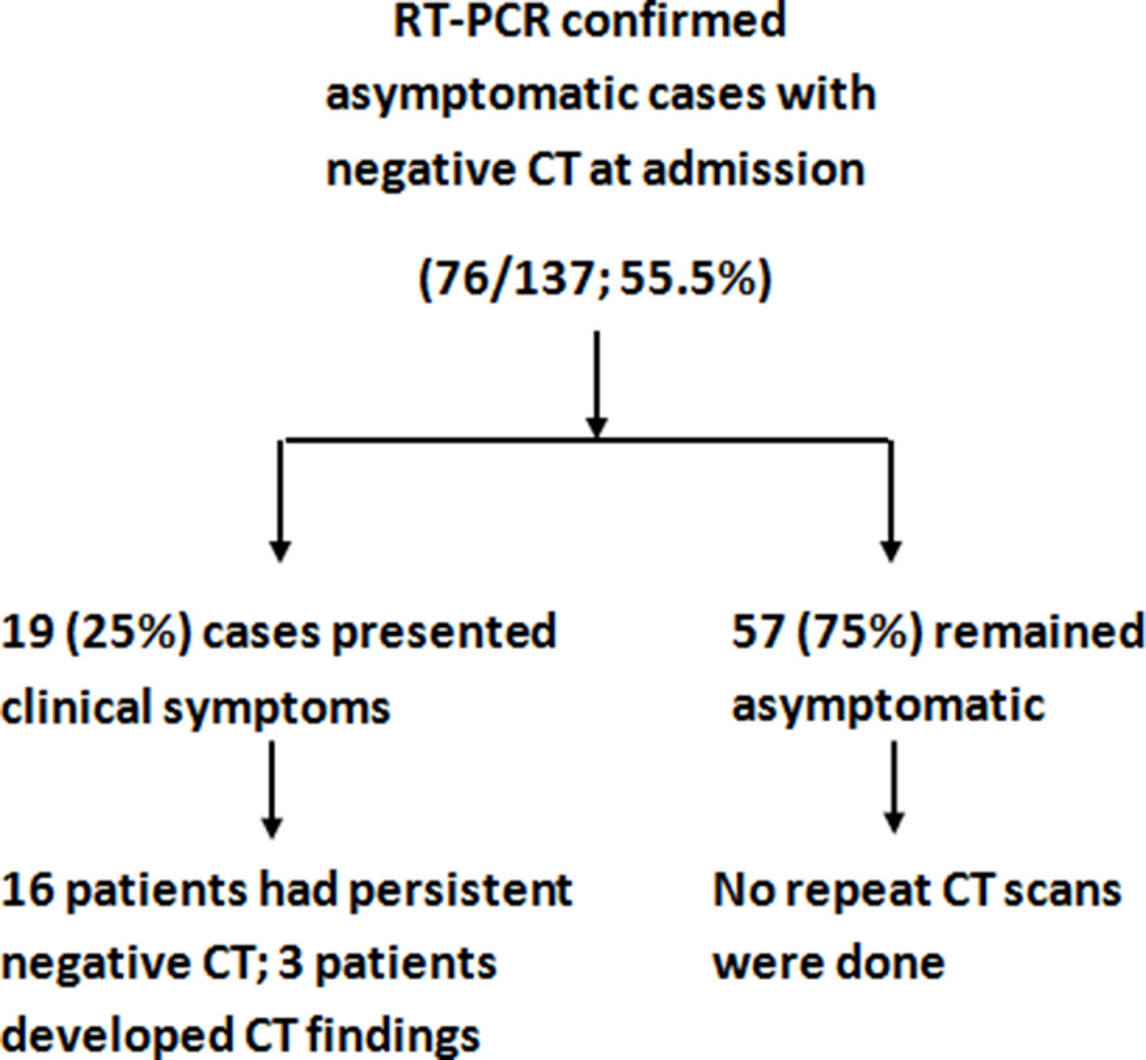
Flowchart depicting the course of asymptomatic COVID-19 cases with a negative chestC T.

Among the total study population of 137 patients, nearly one-half (61/137; 44.5%) had pulmonary findings on chest CT at admission and 76/137 (55.5%) had a normal or negative CT at admission (Figure 1). The clinical course of initial CT negative group is summarized in Figure 2.

**Figure 3.**
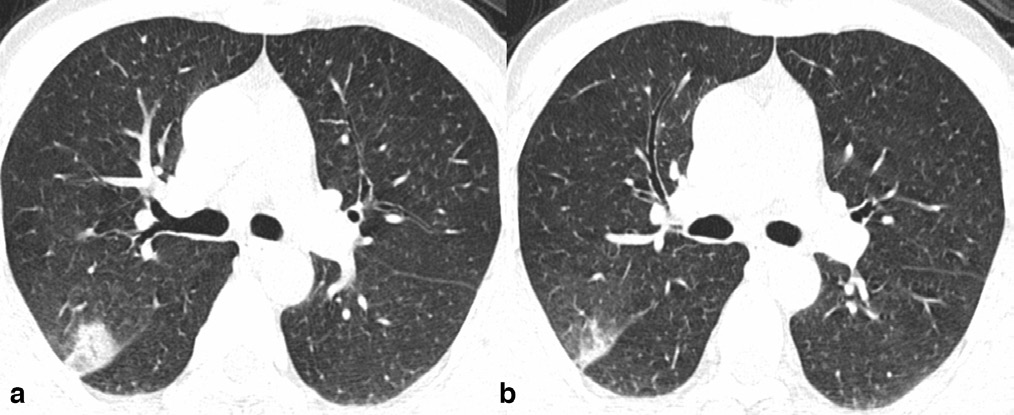
Axialchest CT images in lung window settings of a 46-year-old COVID-19 asymptomaticpatient showing a patch of consolidation in posterior segment of right upperlobe at admission (A). Follow-up CT performed 10 days latter reveal reductionin the size of opacity (B).

**Figure 4.**
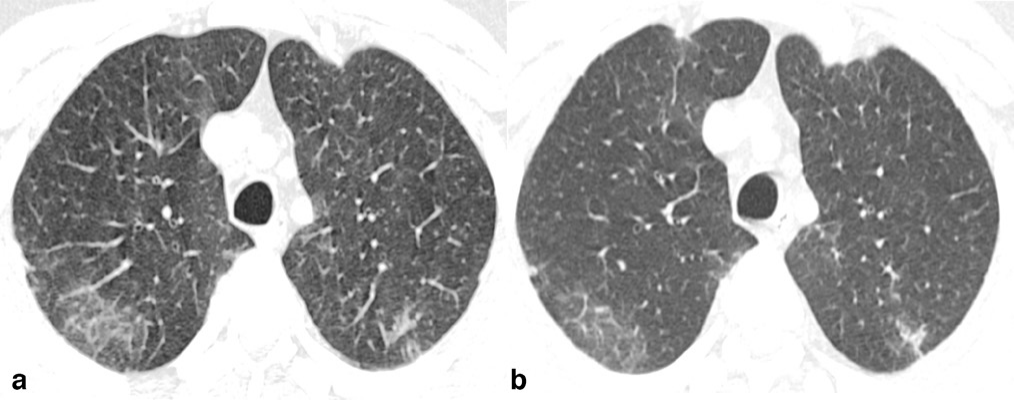
Axialchest CT images in lung window settings of a 51-year-old COVID-19 asymptomaticpatient, obtained 6 days apart, reveal comparable density and similar sizedGGOs with reticulations in subpleural lung in posterior segment of bilateralupper lobes.

**Figure 5.**
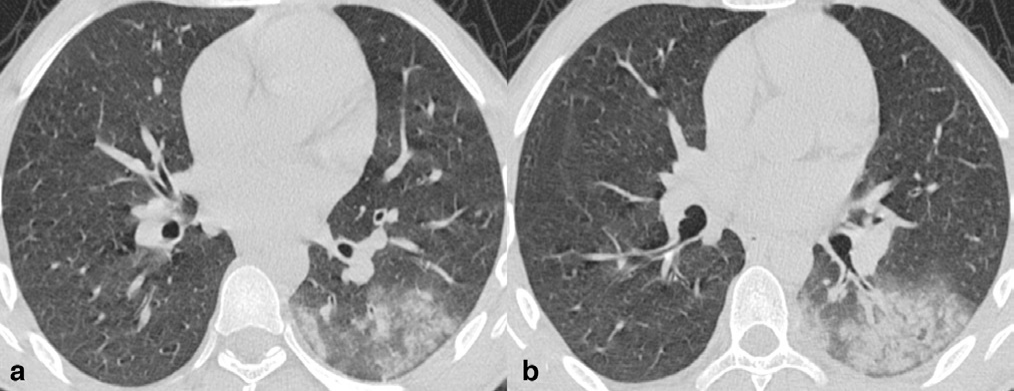
Axialchest CT images in lung window settings of a 29-year-old COVID-19 asymptomaticmale patient showing a GGO with few areas of consolidation in superior segmentof left lower lobe in subpleural location (A). Follow-up CT obtained after agap of 5 days reveals an increase in the size and density of the opacity withtransformation into a frank consolidation (B).

The patients with abnormal CT findings at admission were subdivided into four groups based on the interval change between the first (at admission) and follow-up CT examinations after admission: (a) complete resolution (21/61, 34.4%), (b) partial resolution or improvement group (22/61, 36%) (Figure 3)**,** (c) stable (no change) group (5/61, 8.2%) (Figure 4), (d) worsening/progression group (13/61, 21.4%) (Figure 5). Demographic profile, clinical characteristics and laboratory findings of these groups are summarized in Tables 1 and 2.

**Table 1.** Demographics and clinical characteristics of asymptomatic COVID-19 infected patients

Parameter	Complete lesion resolution	Partial lesion resolution/lesion improvement	Stable (no change)	Lesion worsening/progression	Overall
	(21/61; 34.4%)	(22/61; 36%)	(5/61; 8.2%)	(13/61; 21.4%)	(*n* = 61)
				
Mean age	42.6 ± 13.2	37.7 ± 18.2	41.6 ± 13.6	54 ± 19.7	43.1 ± 17.2
(years) ± SD					

**Gender**					
Male	11	14	3	10	38 (62.3%)
Female	10	8	2	3	23 (37.7%)
**History of exposure**					
Present					
Absent	21 (100%)	22 (100%)	5 (100%)	13 (100%)	61 (100%)
	0	0	0	0	0
**Co-morbid illness**					
None					
Hypertension	19 (90.4%)	19 (86.4%)	0	7 (53.8%)	45 (73.7%)
Diabetes Mellitus	1 (4.7%)	2 (9%)	0	2 (15.4%)	6 (9.8%)
**COPD**	1 (4.7%)	1 (4.5%)	0	1 (7.7%)	3 (4.9%)
**CAD**					
	0	0	0	2 (15.4%)	2 (3.3%)
	0	0	0	1 (7.7%)	1 (1.6%)
**Subsequent symptoms**	0	4 (18.2%)	1 (20%)	9 (69.2%)	14 (22.9%)
**Duration from admission to symptom onset (days)**					
**Fever**	-**-**	4.6 ± 2.98	4.3 ± 2.62	3.1 ± 2.36	4.1 ± 2.2
**Sore throat cough**					
**Fatigue/Malaise**					
**Shortness of breath**					
**Headache diarrhea**	0	3 (13.6%)	1 (20%)	6 (46.1%)	10 (16.4%)
**Dysguesia**	0	1 (4.54%)	0	1 (7.7%)	2 (3.3%)
	0	2 (9.09%)	1 (20%)	4 (30.7%)	7 (11.4%)
	0	1 (4.54%)	1 (20%)	2 (15.4%)	4 (6.6%)
	0			2 (15.4%)	2 (3.3%)
					
	0	2 (9.09%)		4 (30.7%)	6 (9.8%)
	0			1 (7.7%)	1 (1.6%)
	0			1 (7.7%)	1 (1.6%)
**Duration of hospital stay (days)**	15.6 ± 6.61	16.4 ± 5.62	17.1 ± 3.1	27.1 ± 6.3	18.46 ± 6.3
**Deaths**	0	0	0	0	0

CAD, coronary artery disease; COPD, chronic obstructive pulmonary disease.

**Table 2.** Laboratory results of asymptomatic COVID-19 infected patients

Laboratory parameter	Complete lesion resolution	Partial lesion resolution/lesion improvement	Stable (no change)	Lesion worsening/progression	Overall
	(21/61; 34.4%)	(22/61; 36%)	(5/61; 8.2%)	(13/61; 21.4%)	(*n* = 61)
			
Red blood cell count,×10^9^/**L**	4.1 ± 0.17	3.98 ± 0.41	4.0 ± 0.41	3.90 ± 0.47	4.0 ± 0.34
						Total leukocyte count,×10^9^/**L**	5.63 ± 1.19	5.69 ± 1.43	6.1 ± 1.37	3.83 ± 1.02	5.30 ± 1.21
**Absolute neutrophil count,×10^9^/L**	3.33 ± 1.09	3.60 ± 1.44	3.04 ± 1.11	2.58 ± 0.79	3.24 ± 1.31
**Lymphocyte count,×10^9^/L**	1.70 ± 0.31	1.76 ± 0.51	1.81 ± 0.62	1.01 ± 0.29	1.58 ± 0.49
**Platelet count,×10^9^/L**	264.12 ± 62.86	265.8 ± 80.12	276 ± 81.38	211.4 ± 89.76	254.46 ± 77.8
**C-reactive protein, mg/L**	4.86 ± 3.22	8.91 ± 3.89	7.68 ± 4.12	25.61 ± 10.1	10.97 ± 6.03
**Creatinine, μmol/L**	41.30 ± 5.70	42.60 ± 11.11	47.70 ± 15.10	89.13 ± 67.13	52.48 ± 32.1
						**LDH, U/L**	165.42 ± 31.95	171.1 ± 53.65	169.65 ± 53.60	286.0 ± 79.04	193.5 ± 51.9
**D-dimer, mg/L**	0.61 ± 0.21	0.79 ± 1.19	0.71 ± 0.61	0.78 ± 0.96	0.72 ± 0.38

LDH, lactate dehydrogensae.

Among the CT positive group 14/61 (22.9%) patients developed symptoms after admission. The reported symptoms included, fever (10; 71.4%), cough (7, 50%), fatigue/malaise4; 28.6%), shortness of breath (2; 14.3%), headache (6; 42.9%), sore throat (2; 14.3%), diarrhea (1, 1.6%) and dysguesia (1, 1.6%). Two patients who developed shortness of breath required non-invasive oxygen. None of the patients required invasive ventilation. Among the14 patients who developed symptoms, 10 (71.4%) were in lesion progression group, 3 (21.4%) were in the partial resolution or lesion improvement group and 1 (7.1%) was in stable (no change) group. The patients developed symptoms after a mean period of 4.1 ± 2.2 days. The patients in lesion progression group developed symptoms earlier, after a mean of 3.1 ± 2.36 days (Table 3).

**Table 3.** Distribution of lung findings on chest CT in asymptomatic patients

Lung parenchymal abnormalities on CT	Number of patients	%
	(*n* = 137)	
Present	61	44.5
Absent	76	55.5
**Laterality of lung involvement**		
Bilateral	37	60.7
Right lung	13	21.3
Left lung	11	18
**Lobar involvement**		
Right upper lobe	39	63.9
Right middle lobe	23	37.7
Right lower lobe	49	80.3
Left upper lobe	35	57.3
Left lower lobe	54	88.5
**Number of lobes involved**		
Five lobes	1	1.6
Four lobes	3	4.9
Three lobes	9	14.8
Two lobes	14	22.9
One lobe	34	55.8
**Anteroposterior location**		
Anterior	2	3.3
Posterior	52	85.2
Anterior and posterior	7	11.5

Bilateral lung involvement (37; 60.7%) was more common than single lung involvement. In terms of lobar distribution, lower lobes (right 80.3% *vs* left 88.5%) were affected more than the upper lobes. In terms of number of lobes involved, single lobe (34; 55.8%) involvement was more common than ≥2 lobe involvement (Table 3).

GGO was the commonest type of lung opacity, observed in 48/61 (78.7%). GGO with crazy paving pattern was seen in 9 (14.7%) and GGO admixed with patchy consolidation in 2 (3.3%). Pure consolidation was seen in 1 (1.64%). Additional signs observed on CT included intralesional or perilesional segmental or subsegmental pulmonary vessel enlargement in 11 (18%) and subpleural lines in 5 (8.1%). None of the patients showed pleural effusion, pericardial effusion or mediastinal lymphadenopathy (Table 4).

**Table 4.** Type of lung opacities on chest CT

Lung opacity	Number of patients (*n* = 61)	%
GGO	48	78.7
GGO with crazy paving pattern	9	14.7
Pure consolidation	1	1.64
Mixed pattern (GGO with consolidation)	2	3.3
Sub pleural linear/curvilinear lines	5	8.1
Nodules	1	1.64
Reticulations	1	1.64
Halo sign	1	1.64
Segmental vessel enlargement	11	18
Bronchial wall thickening	3	4.9
Bronchial dilatation	1	1.64
Air bronchogram sign	3	4.9
Air bubble sign	2	3.3

GGO, ground glass opacity.

Comparison between the admission CT and follow-up CT during the course of hospitalization revealed evolution of CT findings in 13 (21.4%) patients. The evolution of lesions included increase in the size of opacity, involvement of other lung lobes and increase in the density of lung opacities in the form of progression of GGO into crazy paving pattern or formation of consolidation. The patients in progression group (54 ± 19.7 years) were older and had higher frequency of co-morbidities (46.2%) compared to the other three groups (10.4%). The patients in progression group had a significantly higher C-reactive protein (*p* = 0.029), higher lactate dehydrogenase (*p* = 0.002) and lower lymphocyte count (*p* = 0.008) at the time of admission than the other groups. The average hospital stay of 27.1 ± 11.4 days in the progression group was significantly longer than others (*p* = 0.016) (Table 1). All the patients recovered and were discharged at the time of writing of this manuscript.

## Discussion

SARS-CoV-2, a single-stranded RNA virus belonging to the family of betacorona viruses is the culprit virus responsible for the ongoing pandemic of COVID-19. SARS-CoV-2 is believed to have originated from bats which act as the natural reservoir. The disease spreads through human-to-human contact via respiratory route.^15^ The clinical manifestations of the disease vary from no symptoms to mild symptoms to severe illness and death. There are limited data available on the combined clinical and imaging follow-up of asymptomatic cases.

In the present study, 61 (44.5%) asymptomatic cases had abnormal lung findings on chest CT. It has been observed that asymptomatic cases can have a positive CT. Inui et al^7^ reported 56% of asymptomatic COVID-19 cases with abnormal lung findings in Diamond Princess Cruise Ship. Bandirali et al^16^ reported pulmonary parenchymal abnormalities in 59% (100/170) of asymptomatic or minimally symptomatic patients. Multiple other cases of asymptomatic COVID-19 patients with pulmonary findings consistent with COVID-19 have been reported.^17^ The converse has also been reported where symptomatic cases can have a negative CT.^7,18^

The distribution and type of pulmonary opacities in asymptomatic cases may resemble the CT findings in symptomatic cases.^18–21^ However, asymptomatic and mildly symptomatic cases have a lower percentage of lung involvement with low CT severity score. It has been widely reported that the percentage of the total lung involvement signifying the disease burden determines the severity of the disease and the final clinical outcome.^21,22^ Inui et.al^7^ in the famous Diamond Princess Cruise Ship made a comparison of total CT score (determined visually as the percentage of total lung involvement) and found a significantly lower CT score in asymptomatic cases compared to the symptomatic cases (*p*-value < 0.05). They also reported that consolidations were more common in symptomatic cases (41%) compared to asymptomatic cases (17%), whereas GGOs predominated in asymptomatic cases (83% *vs* 59%). We observed GGOs in 93.4% asymptomatic cases whereas consolidation was observed in only 4.94%.

Parry et al^21^ reported that the percentage of lung opacification is a surrogate of clinical outcome in COVID-19 pneumonia with a higher percentage of lung involvement suggesting an adverse outcome. Similarly, Tabatabaei et al^22^ also reported that the percentage of total lung involvement determines the severity of the disease.

Imaging follow-up of the clinically asymptomatic cases with abnormal lung findings at admission revealed almost all possible changes in lung opacities which included, complete resorption (34.4%), partial resorption or improvement (36%), stable lesion (no change) (8.2%) and worsening or progression (21.4%).

The patients in progression group were older and had a significantly higher C-reactive protein, higher lactate dehydrogenase and lower lymphocyte count at the time of admission than the other groups. Older age, co-morbidities, lower lymphocyte count, higher CRP and LDH seem to represent the potential risk factors leading to clinicoradiological progression. Yu et al^23^ in their study reported that age, presence of co-morbidities, low lymphocyte count, presence of consolidations, crazy-paving pattern, larger size of pulmonary opacities and pleural effusion were associated with severe illness. Older age has been found to an important risk factor for severe disease and adverse outcome.^18,21,22^

Yang et al in their study reported that asymptomatic patients were younger (median age of 37 years) compared to symptomatic patients (56 years) (*p* < .001) and had a higher CD4 +T lymphocyte count and showed a faster lung recovery on CT scans (9 *vs* 15 days) (*p* = .003)^24^

Our results corroborate the clinical and imaging findings in asymptomatic cases reported by these studies. However, in view of small number of patients in the progression group in our study further clinical studies with larger sample sizes may be undertaken to validate the results of our study.

Intralesional or perilesional segmental or subsegmental vascular enlargement was observed in 18% of cases. This is a unique finding which has not been reported earlier in any infectious pneumonia. This intriguing vascular finding can have a diagnostic value. The presence of intralesional vascular enlargement can differentiate COVID-19 pneumonia from other causes of infectious pneumonia. Though, the exact pathophysiological mechanism underpinning this intralesional vascular enlargement is unclear at present but it has been suggested that three possible mechanisms could account for this finding.^25^ Cascading effect of inflammatory cytokines may result in intralesional vascular enlargement. Alternately, microvascular thrombosis (immunothrombosis) has also been suggested as the underlying cause.^26^

Though CT has helped us in the understanding of the disease but the guidelines issued by various radiological societies do not recommended CT as a screening or diagnostic tool in lieu of nucleic acid testing for COVID-19 pneumonia. European Society of Radiology and the European Society of Thoracic Imaging do not recommend performance of CT in asymptomatic or mildly symptomatic COVID-19 patients. According to the joint statement of European Society of Radiology and European Society of Thoracic ImagingI, CT should be reserved for the evaluation of patients with severe respiratory symptoms such as dyspnoea and desaturation. However, in selected circumstances CT may also be helpful in patients with milder symptoms who have co-morbidities, such as diabetes, obesity, chronic respiratory disease, etc.^27^ Repeat CTs are not indicated in patients that are recovering. However, a repeat examination may be indicated in cases with suspected complications (*e.g.* superinfection, pulmonary embolism).^27^

According to American College of Radiology guidelines, CT should be reserved for hospitalized, symptomatic patients with specific clinical indications like deteriorating respiratory status.^28^

There are a few limitations to this study. First, there may have been a selection bias as imaging was performed in non-consecutive asymptomatic cases. Second, the small size of study population especially lesser number of patients in the progression group is also a limitation.

## Conclusion

In conclusion, asymptomatic cases with COVID-19 pneumonia have abnormal lung findings on CT. The clinicoradiological course of these asymptomatic cases is variable. Clinically, some recover without developing symptoms, some present few mild symptoms and others deteriorate. Similarly, imaging follow-up may reveal resolution (partial or complete), progression or no change. Older age, lower lymphocyte count, higher CRP and LDH and presence of co-morbidities are more commonly associated with clinicoradiological progression of the disease.
